# Energy Values and Standardized Ileal Digestibility of Amino Acid in Fermented Cottonseed Meal Fed to Growing Pigs

**DOI:** 10.3390/ani16142137

**Published:** 2026-07-09

**Authors:** Zhiheng Shu, Weikun Li, Li Wang, Xue Hua, Jun Fang, Gang Liu, Hao Yao, Jie Cao, Beibei He, Hongmei Jiang

**Affiliations:** 1 College of Bioscience and Biotechnology, Hunan Agricultural University, Changsha 410128, China; shuzhiheng@stu.hunau.edu.cn (Z.S.);; 2 Academy of National Food and Strategic Reserves Administration, Beijing 100037, China; 3 Board of Directors Department, Changsha IMADEK Intelligent Technology Company Limited, Changsha 410137, China; 4 Nanjing Institute of Agricultural Mechanization, Ministry of Agriculture and Rural Affairs, Nanjing 210014, China; 5 School of Chemistry and Materials Science, Hunan Agricultural University, Changsha 410128, China

**Keywords:** fermented cottonseed meal, growing pigs, energy, amino acid digestibility

## Abstract

To evaluate the nutritional value of fermented cottonseed meal (**FCSM**) for growing pigs, ten representative FCSM samples were collected and assessed in two experiments for the standardized ileal digestibility (**SID**) of amino acids (**AAs**) and for digestible energy (**DE**) and metabolizable energy (**ME**). In **Experiment 1**, 11 growing pigs (Duroc × Landrace × Yorkshire, 25 ± 1.8 kg) fitted with a T-cannula were assigned to an 11 × 6 incomplete Latin square design, with 11 diets (one nitrogen-free diet and 10 diets containing FCSM as the sole nitrogen source) and 6 periods. All diets contained 0.30% Cr_2_O_3_ as an indigestible marker. **Experiment 2** used 22 healthy crossbred barrows with an initial body weight of 45 ± 2.3 kg. A replicated 11 × 3 incomplete Latin square design was adopted, comprising 11 dietary treatments and 3 periods. The dietary treatments included a corn–soybean meal basal diet (control) and 10 FCSM-containing diets. The results demonstrated marked variation among FCSM samples in terms of chemical composition and energy content. The mean Ca/P ratio was 0.32. The average DE and ME were 11.20 MJ/kg (range 9.69–12.65 MJ/kg) and 10.68 MJ/kg (range 8.55–11.84 MJ/kg), respectively. The SID values for Lys, Met, Thr, Trp and Val were 65.90% (60.01 to 68.92%), 77.34% (74.48 to 81.78%), 68.10% (59.97 to 72.02%), 72.12% (70.25 to 73.62%), and 75.50% (68.60 to 78.65%), respectively. Stepwise regression analysis generated prediction equations for estimating DE and ME from chemical composition. These data fill a gap in energy and amino acid digestibility parameters for FCSM, provide a basis for precision diet formulation, and indicate the need to supplement limiting amino acids and optimize the Ca/P ratio.

## Simple Summary

Microbial fermentation reduces anti-nutritional factors and improves the nutritional quality of cottonseed meal, yet the nutritional value of fermented cottonseed meal (**FCSM**) for growing pigs has not been systematically evaluated. To address this gap, ten representative FCSM samples were collected from commercial producers and research institutions across China and analyzed for energy content, nutrient digestibility, and chemical composition. Substantial variation in digestible energy, metabolizable energy, and chemical profiles was observed among the samples. Based on the chemical composition, regression models were developed to predict energy values. This study provides a reference for the precise incorporation of FCSM into growing pig diets.

## 1. Introduction

The shortage of protein feed resources has become a critical bottleneck restricting the sustainable development of livestock production [1]. Cottonseed meal (**CSM**), a by-product obtained after dehulling, delinting, and oil extraction of cottonseeds, contains 30–50% crude protein but also harbors anti-nutritional factors such as free gossypol, tannins, and phytate [2]. Moreover, although CSM is rich in amino acids, its imbalanced amino acid profile further limits its feeding value [3].

Microbial fermentation effectively reduces free gossypol and tannin contents, produces beneficial metabolites such as short-chain fatty acids and small peptides, and thereby substantially improves the nutritional quality of CSM [4]. Fermented cottonseed meal (**FCSM**) is therefore regarded as a promising alternative plant protein source with the potential to alleviate the shortage of high-quality protein feed in China. To date, studies on replacing soybean meal with FCSM have largely focused on poultry and ruminants, with emphasis on growth performance, fat deposition, and economic returns [5,6]. In growing pigs, existing work has primarily addressed the effects on gut microbiota, serum biochemical indices, and growth performance [7], whereas systematic data on digestible energy (**DE**) and metabolizable energy (**ME**) of FCSM for growing pigs remain scarce.

To this end, the present study collected representative FCSM products from commercial producers and research institutions across China, determined their DE and ME values and the standardized ileal digestibility (**SID**) of amino acids (**AAs**) in growing pigs, and further developed prediction models. The results provide baseline data for the precise incorporation of FCSM into pig diets.

## 2. Materials and Methods

The experimental animals were purchased from Liuyang Annong Technology Comprehensive Development Co., Ltd., Changsha, China. The animal experiments in this study were conducted at the Digestive and Metabolic Laboratory, Institute of Subtropical Agriculture, Chinese Academy of Sciences. All experimental procedures were reviewed and approved by the Biomedical Research Ethics Committee of Hunan Agricultural University (Approval No. 2023-165).

### 2.1. Fermented Cottonseed Meal Samples

The ten FCSM samples used in this study were collected from major cotton-producing and processing regions in China, covering the mainstream aerobic, anaerobic, and sequential fermentation processes, and were sourced from leading domestic producers and national research institutions (Table 1).

### 2.2. Experimental Design and Sample Collection

#### 2.2.1. Experiment 1: Determination of AA Digestibility

Eleven healthy barrows (Duroc × Landrace × Yorkshire, 25 ± 1.8 kg) were selected, and a T-cannula was surgically installed at the distal ileum according to the method of GB/T 40830-2021 [8], followed by a 14-day recovery period. The pigs were arranged in an 11 × 6 incomplete Latin square design, with 11 diets (one nitrogen-free diet and 10 diets containing FCSM as the sole nitrogen source; composition shown in Table 2 and Table 3) and 6 periods. All diets were formulated to meet NRC (2012) requirements and contained 0.30% Cr_2_O_3_ as an indigestible marker [9]. Pigs were individually housed in metabolism cages at 25 ± 2 °C with free access to water and received routine disinfection and immunization. They were fed twice daily at 08:30 and 17:30. Each experimental period consisted of a 5-day adaptation phase and a 2-day ileal digesta collection phase. Ileal digesta were collected into plastic bags and immediately stored at −20 °C. At the end of the experiment, digesta samples from each pig were thawed, pooled, and sub-sampled following the procedure described by Pan [10].

#### 2.2.2. Experiment 2: Determination of DE and ME

Twenty-two healthy crossbred barrows (Duroc × Landrace × Yorkshire, initial body weight 45 ± 2.3 kg) were used in a replicated 11 × 3 incomplete Latin square design, with 11 dietary treatments and 3 periods. The dietary treatments consisted of a corn–soybean meal basal diet (control) and 10 FCSM diets (groups 1–10). All diets were formulated to meet the nutrient requirements for growing-finishing pigs according to NRC (2012) [9], and the dietary composition is detailed in Table 4 and Table 5. Feeding management was the same as in Experiment 1. Each period lasted 12 days, comprising a 7-day adaptation phase and a 5-day total fecal and urine collection phase. Pigs were weighed before each period, and the feed allowance for the subsequent period was set at 4% of the average body weight. For fecal samples, 10 mL of 10% sulfuric acid was added per 100 g of feces for nitrogen fixation, and after thorough mixing, 400–600 g of feces were dried in an oven at 65 °C for 72 h, equilibrated for 24 h, weighed, ground to pass a 40-mesh sieve, and stored at −20 °C for subsequent physicochemical analysis. For urine samples, 50 mL of 6 mol/L HCl was added to each collection bucket, and after mixing and filtration, a 10% aliquot of the total urine was stored at −20 °C for subsequent physicochemical analysis.

### 2.3. Chemical Analysis

Samples were analyzed in accordance with the Chinese national standard methods for the following components: crude protein (**CP**, GB/T 6432-2018), dry matter (**DM**, GB/T 6435-2014), ether extract (**EE**, GB/T 6433-2025), crude ash (Ash, GB/T 6438-2025), crude fiber (**CF**, GB/T 6434-2022), neutral detergent fiber (**NDF**, GB/T 20806-2022), calcium (**Ca**, GB/T 6436-2018), phosphorus (**P**, GB/T 6437-2018), dietary fiber (insoluble dietary fiber, **IDF**; soluble dietary fiber, **SDF**; total dietary fiber, **TDF**; GB 5009.88-2023), minerals (Cu, Fe, Mg, Mn, K, Na, Zn; GB/T 13885-2017), I and Se (GB/T 14924.12-2001), AAs (GB/T 18246-2019), tryptophan (**Trp**, GB/T 15400-2018), and Cr (GB/T 13088-2006) [11,12,13,14,15,16,17,18,19,20,21,22,23,24]. Acid detergent fiber (**ADF**) was determined following NY/T 1459-2022, and total starch (**TS**) was measured according to AOAC 996.11 [25,26]. Gross energy (**GE**) was determined using a fully automated oxygen bomb calorimeter (Model 5E-AC8018, Changsha Kaide Measurement and Control Instruments Co., Ltd., Changsha, China) [27].

### 2.4. Calculations and Statistical Analyses

Dietary DE and ME in Experiment 1 were calculated following the method of Adeola [28]. Ileal endogenous losses of AAs (**IAA**), apparent ileal digestibility (**AID**), and standardized ileal digestibility (**SID**) were determined according to the procedures described by Wang et al. [29]. All data were first compiled in Microsoft Excel 2019 and subsequently analyzed using the MIXED procedure of SAS 9.4 (SAS Institute Inc., Cary, NC, USA). Pearson correlation coefficients were computed with the CORR procedure to examine relationships between chemical composition and DE, ME, and SID. Prediction equations were developed using the REG procedure. Significance was declared at *p* < 0.05, and trends were considered at 0.05 < *p* < 0.10. Graphs were generated with the ggplot2 package (version 3.3.3).

## 3. Results

### 3.1. Chemical Composition and AA of FCSM

As shown in Table 6, the coefficients of variation (**CV**) for DM, GE, CP, Ash, and TDF among the 10 FCSM samples were all below 10%, with those of CP and TDF approaching 10%. The CVs for EE, CF, TS, SDF, IDF, NDF, and ADF all exceeded 10%. Notably, the CV of SDF was greater than 100%, and the SDF content of sample No. 5 was approximately four times the mean and ten times the lowest value.

The amino acid composition of the FCSM samples is presented in Table 7. The CVs of Thr, Leu, Val, and Asp were close to 10%, while those of the remaining amino acids exceeded 10%. Glutamic acid (Glu) was the most abundant, with a mean content of 9.46%, followed by Arg (4.8%), Asp (4.33%), and Leu (2.66%). Met, Trp, and Cys showed the lowest concentrations, averaging only 0.48%, 0.56%, and 0.23%, respectively.

Mineral composition is shown in Table 8, and overall variation was substantial. Among the macro-minerals, K content was the highest (1.39%) and Na content the lowest (0.11%). Average Ca content was 0.28% and P was 0.92%, yielding a mean Ca-to-P ratio of 1:3.17. For trace minerals, Fe had the highest mean concentration (784.01 mg/kg) with a CV of 73.47%; Zn (54.38 mg/kg), Cu (7.43 mg/kg), and Mn (47.38 mg/kg) showed CVs of 24.38%, 21.37%, and 58.78%, respectively. I (5.33 mg/kg) and Se (0.18 mg/kg) had CVs of 22.21% and 56.02%, respectively.

The anti-nutritional factor contents varied considerably among the FCSM samples (Table 9). The mean concentrations of free gossypol (**FG**), phytate, and tannins were 0.33 mg/g, 0.75%, and 12.74 mg/g, respectively.

### 3.2. The SID of AA in FCSM

The SID of AAs among the 10 FCSM samples showed limited overall variation, with significant differences detected only for Thr, Val, Ala, and Pro (*p* < 0.05) (Table 10). Among the essential amino acids, Arg had the highest mean SID at 89.97% (87.25 to 91.62%), while Lys had the lowest at 65.90% (60.01 to 68.92%). The mean SID values for Met, Thr, Trp, Ile, Leu, Val, His, and Phe were 77.34%, 68.10%, 72.12%, 72.40%, 76.94%, 75.50%, 77.64%, and 82.61%, respectively. Among the non-essential amino acids, Glu exhibited the highest mean SID at 86.89% (85.56 to 87.82%), whereas Ala showed the lowest at 72.08% (67.11 to 78.69%). Additionally, the mean SID of total amino acids (TAA) was 79.89% (71.61 to 81.52%).

### 3.3. DE and ME Contents of FCSM

As shown in Table 11, the DE and ME of the 10 FCSM samples for growing pigs varied considerably, with CVs of 16.64% and 10.51%, respectively. The average DE was 11.20 MJ/kg (9.69 to 12.65 MJ/kg), and the average ME was 10.68 MJ/kg (8.55 to 11.84 MJ/kg). The mean ME/DE ratio was 0.94 (0.88 to 0.98).

### 3.4. Correlation and Prediction Equations for DE and ME

**Figure 1:**
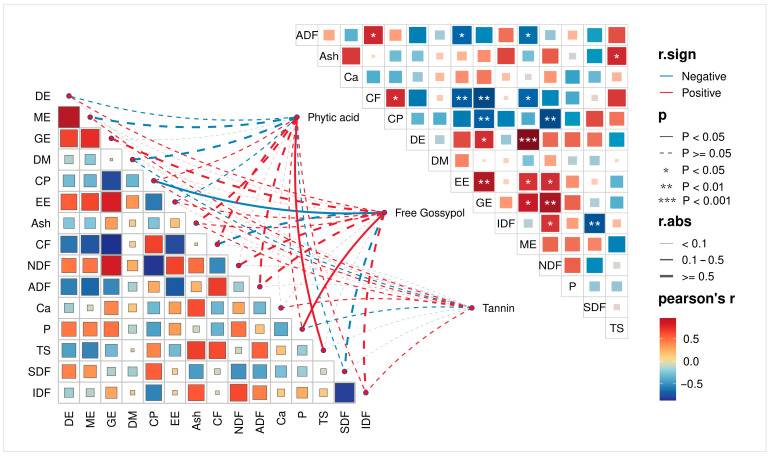
Correlation network heatmap of the chemical composition, DE, ME, and anti-nutritional factors in FCSM.

As shown in Figure 1, DE was positively correlated with ME (*p* < 0.01) and with GE (*p* < 0.05). ME showed positive correlations with GE and EE (*p* < 0.05) and negative correlations with CF and ADF (*p* < 0.05). GE was positively correlated with EE and NDF (*p* < 0.01) and negatively correlated with CP and CF (*p* < 0.01). EE exhibited a negative correlation with CF (*p* < 0.01) and with ADF (*p* < 0.05), and a positive correlation with NDF (*p* < 0.05). CP was negatively correlated with NDF (*p* < 0.01) and positively correlated with CF (*p* < 0.05). CF showed a positive correlation with ADF (*p* < 0.05). SDF was negatively correlated with IDF (*p* < 0.01). Phytic acid was positively correlated with TS (*p* < 0.05). Free gossypol showed a negative correlation with CP (*p* < 0.05) and a positive correlation with P (*p* < 0.05).

The regression equations for DE and ME of FCSM are presented in Table 12. The best-fit equations were: DE [MJ/kg DM] = 0.53 × GE − 0.10 × SDF + 1.70 (R^2^ = 0.72, *p* < 0.05), ME [MJ/kg DM] = 1.03 × DE − 0.94 (R^2^ = 0.91, *p* < 0.01).

## 4. Discussion

### 4.1. Chemical Composition of FCSM Samples

Ten FCSM samples were collected from five provinces in this study, covering aerobic, anaerobic, and combined aerobic–anaerobic fermentation processes, and are therefore highly representative. The average CP content of the 10 FCSM samples was 46.86% (range: 41.10–55.2%), which falls within the CP range for cottonseed meal specified by NRC (2012) [9] and is consistent with the trend of increased CP content in fermented cottonseed meal reported in the literature [30].

Dietary fiber (**DF**) was traditionally considered an anti-nutritional factor in conventional nutrition research because it resists degradation by endogenous digestive enzymes and may reduce nutrient digestibility [31]. With advances in research, the beneficial role of DF in gut health has become widely recognized [32]. Although pigs cannot directly utilize DF via their own digestive enzymes, the gut microbiota can ferment DF into lactate and short-chain fatty acids (**SCFAs**), which are closely linked to host intestinal health [33,34]. Of the fiber fractions, SDF is more readily fermented than IDF [35]. In the present study, the CV for EE, TS, and the fiber components (CF, IDF, NDF, ADF) all exceeded 10%, and those of IDF, NDF, and ADF were above 15%, while the CV for SDF reached as high as 109.8%. Further analysis showed that the SDF content of sample No. 5 was approximately four times the mean and ten times the lowest value, and that sample No. 5 was the only one produced by an aerobic-anaerobic sequential fermentation process. Given the potential advantages of this process observed in the present study, we hypothesize that aerobic–anaerobic sequential fermentation may promote SDF enrichment while reducing IDF content; however, this hypothesis requires verification with additional replicate samples.

The CVs of amino acids in the 10 samples were all close to or above 10%, which may be attributable to differences in fermentation substrates, strains, and conditions [36,37]. Intramuscular fat (**IMF**) is a key determinant of meat quality, particularly tenderness, flavor, and juiciness [38]; however, greater IMF content is usually accompanied by reduced carcass leanness and increased backfat thickness [39]. Dietary supplementation with Glu and Arg has been shown to increase IMF, improve meat color and fatty acid composition, and enhance meat quality without affecting growth performance or increasing subcutaneous fat in pigs [40]. In the 10 FCSM samples analyzed in this study, Glu was consistently the most abundant amino acid, averaging 9.46%, followed by Arg (4.8%), both of which exceeded the corresponding levels in soybean meal (NRC 2012) [9]. It is therefore speculated that FCSM may have the potential to improve pork quality, though further experiments are needed to verify this.

The concentrations of limiting amino acids in the 10 FCSM samples were generally low; for example, the average contents of Met, Trp, and Cys were only 0.48%, 0.56%, and 0.23%, respectively, which may restrict animal growth [41]. In addition, mineral composition varied considerably and the Ca/P ratio was low, likely resulting from differences in production regions, cottonseed varieties, and processing techniques [42]. Therefore, in practical diet formulation, ingredient combinations should be optimized to compensate for the inadequate supply of limiting amino acids and the low calcium-to-phosphorus ratio.

Regarding anti-nutritional factors, the FG content of all samples complied with the feed hygiene standard (GB 13078-2017) [43]. The mean contents of FG, Phytic acid, and Tannins were all lower than those in cottonseed meal, consistent with the reported trend of microbial degradation [44,45].

### 4.2. Digestibility of Amino Acids

Accurate determination of the SID of AAs is essential for the precise supply of amino acids in pig diets [46]. After correcting for endogenous losses, the mean SID values of Lys, Met, Thr, Trp, and Val in FCSM were 65.90%, 77.34%, 68.72%, 71.81%, and 75.73%, respectively. These values are slightly higher than the SID of cottonseed meal reported in NRC (2012) [9] and follow a similar pattern [9]. Because of its high moisture content, fermented cottonseed meal is typically dried for storage after fermentation. The elevated temperatures during fermentation and, in particular, during this drying step may induce Maillard reactions, which preferentially affect amino acids with reactive ε-amino groups such as lysine, thereby reducing their digestibility [47,48]. This likely explains why the SID of amino acids did not increase as markedly after fermentation as anticipated.

Furthermore, in contrast to previous studies reporting large variation in the SID of most amino acids among cottonseed meal from different sources [49,50], the present study observed relatively small differences in amino acid digestibility among the fermented products. This may be explained by a homogenization effect of fermentation: the process reduces anti-nutritional factors such as free gossypol and phytate [45], disrupts cell wall fiber structures, and degrades macromolecular proteins into small peptides and free amino acids [30,51], thereby largely eliminating the variation in digestibility caused by differences in anti-nutritional factor content, physical barriers, and protein structure among cottonseed meal sources. This process converges the nutritional quality of the raw material to a relatively uniform level, such that the SID of most amino acids no longer differs significantly among samples. However, the possibility remains that the limited sample size resulted in insufficient statistical power to detect true differences, and this requires further investigation.

### 4.3. Energy Content of FCSM 

DE and ME are critical energy parameters in pig diet formulation [52]. To date, systematic data on the available energy content of FCSM for growing pigs remain limited. In this study, the lowest DE and ME values of the FCSM samples were still higher than the corresponding values reported for cottonseed meal in NRC (2012) [9]. This may be attributed to multiple effects of microbial fermentation. First, it degrades anti-nutritional factors such as free gossypol and phytate, relieving their inhibitory effect on digestive enzymes and thereby enhancing nutrient digestibility [53,54]. Second, it disrupts the lignocellulosic complex and promotes the conversion of insoluble fiber to soluble fiber, which is more readily fermented by hindgut microbes into short-chain fatty acids; this in turn lowers intestinal pH, suppresses the proliferation of pathogenic bacteria, optimizes the microbial community structure, and improves gut health [55,56]. Third, it releases encapsulated nutrients, breaks down macromolecular proteins, and dismantles phytate–nutrient chelates, further improving the efficiency of digestion and absorption [57,58,59]. Together, these mechanisms ultimately elevate the available energy value of fermented cottonseed meal.

Correlation analysis (Figure 1) showed that DE was significantly and positively correlated only with ME and GE (*p* < 0.05). Based on GE, prediction Equation (1) for DE was developed (Table 12); the overall *p* value of 0.04 reached significance, but the coefficient of determination (R^2^) was 0.44, indicating only moderate model fit and limited prediction accuracy. Therefore, variables showing trends toward significance (0.05 < *p* < 0.10) were introduced into a stepwise regression analysis, yielding the best-fit prediction Equation (2), in which SDF was identified as a positive contributor—consistent with our earlier speculation. Stepwise regression further revealed that DE was the best predictor of ME, and Equation (3) was thus established. To enhance the practical value of the model, DE was removed and another stepwise regression was conducted, resulting in Equation (4). However, given the limited sample size, the proposed equations need to be validated with a larger independent dataset. Consistent with most studies, IDF and ADF remained negative contributors to DE and ME in FCSM in the present study [49,60,61]. This suggests that, at the current fermentation level, a large proportion of the fiber is still not sufficiently degraded into energy substrates that can be efficiently utilized by pigs. In biofuel research, pretreating lignocellulosic biomass with bacteria or fungi before anaerobic digestion has been widely applied [62,63,64]. Therefore, for highly lignocellulosic feedstocks such as cottonseed meal, adopting an aerobic-anaerobic sequential fermentation process similar to that used for sample No. 5 may represent a feasible approach in pig feed manufacturing, although further studies are needed for verification.

## 5. Conclusions

The present study evaluated the nutritional value of FCSM derived from different sources and fermentation processes for growing pigs. Substantial variation was observed among samples in proximate nutrients, amino acids, and fiber fractions. Moreove, limiting amino acid concentrations were low and the Ca/P ratio was suboptimal, requiring adjustments in practical diet formulation. In addition, prediction equations for DE and ME were preliminarily developed, though their accuracy needs to be further validated with additional data. Overall, this study fills a gap in the fundamental nutritional parameters of FCSM for growing pigs and provides a scientific basis for its precise application in pig diets.

## Figures and Tables

**Table 1:** Fermented cottonseed meal sample collection information.

No.	Sample Source	Strains	Fermentation Process	Location in China
1	Xinjiang Hipro Bio-Tech Co., Ltd., Wujiaqu, China.	*Saccharomyces*, *lactic acid bacteria*, *Bacillus*	Aerobic	Xinjiang
2	Jize County Tiande Biological Feed Co., Ltd., Handan, China.	*Saccharomyces*, *lactic acid bacteria*, *bacillus subtilis*	Aerobic	Hebei
3	Shengliyuan Biotechnology Co., Ltd., Qingdao, China.	*Candida utilis*, *Aspergillus oryzae*	Aerobic	Shandong
4	Academy of National Food and Strategic Reserves Administration., Beijing, China.	*Saccharomyces*, *lactic acid bacteria*, *bacillus subtilis*	Aerobic	Beijing
5	Zhejiang Kefeng Biological Technology Co., Ltd., Jiaxing, China.	*Saccharomyces*	autoclaving → Aerobic → Anaerobic	Zhejiang
6	Handan Jimuyuan Biological Feed Co., Ltd., Handan, China.	*Saccharomyces*, *lactic acid bacteria*, *bacillus subtilis*	Aerobic	Hebei
7	Xinjiang Taikun Group Co., Ltd., Changji, China.	*Bacillus coagulans*, *Candida utilis*, *Aspergillus niger*	Aerobic	Xinjiang
8	Xinjiang Hipro Bio-Tech Co., Ltd., Wujiaqu, China.	*Saccharomyces*, *bacillus subtilis*	Anaerobic	Xinjiang
9	Academy of National Food and Strategic Reserves Administration, Beijing, China.	*Saccharomyces*, *lactic acid bacteria*, *bacillus subtilis*	Anaerobic	Beijing
10	Academy of National Food and Strategic Reserves Administration, Beijing, China.	*Saccharomyces*, *lactic acid bacteria*, *bacillus subtilis*	Enzyme preparation & Anaerobic fermentation	Beijing

**Table 2:** Ingredient composition of diets in Experiment 1 (as-fed basis, %).

Ingredients	NF Diet ^1^	FCSM Diet ^2^
FCSM	-	40.00
Cornstarch	78.80	43.20
Sucrose	10.00	10.00
Soybean oil	3.00	3.00
Carboxymethyl cellulose	4.00	-
Monocalcium phosphate	2.00	2.00
Limestone	0.70	0.70
Sodium chloride	0.30	0.30
Potassium carbonate	0.30	-
Magnesium oxide	0.10	-
Vitamin and mineral Premix ^3^	0.50	0.50
Cr_2_O_3_	0.30	0.30
Total	100.00	100.00

^1^ nitrogen-free diet. ^2^ Ten samples of fermented cottonseed meal (FCSM) were included in the diets at an identical substitution ratio. ^3^ The premix provides the following per kg of diets: VA 4200.00 IU, VB 12.00 ug, VB1 3.40 mg, VB_2_ 5.63 mg, VB_3_ 28.00 mg, VB5 20.50 mg, VB_6_ 2.70 mg, VD_3_ 400.00 IU, VE 36.00 IU, VK_3_ 1.20 mg, VH 0.18 mg, ChCl 1.00 g, FA 0.80 mg, MnSO_4_ 40.00 mg, FeSO_4_ 70.00 mg, ZnSO_4_ 70.00 mg, CuSO_4_ 70.00 mg, IK 0.30 mg, Na_2_SeO_3_ 0.30 mg.

**Table 3:** Amino acid composition of diets in Experiment 1 (as-fed basis, %).

Items	FCSM Diet No.										Mean	CV %
	1	2	3	4	5	6	7	8	9	10		
Dry matter	92.37	93.00	91.83	92.94	91.96	90.25	92.72	90.64	90.36	90.27	91.63	1.25
Essential AA												
Lys	0.56	0.46	0.53	0.55	0.72	0.59	0.58	0.57	0.58	0.56	0.57	11.31
Met	0.17	0.20	0.17	0.19	0.24	0.16	0.20	0.17	0.17	0.20	0.19	12.87
Thr	0.56	0.54	0.59	0.58	0.7	0.51	0.55	0.56	0.51	0.55	0.57	9.56
Trp	0.21	0.15	0.21	0.20	0.24	0.17	0.24	0.24	0.25	0.24	0.22	15.70
Ile	0.6	0.56	0.62	0.59	0.72	0.53	0.55	0.58	0.51	0.53	0.58	10.44
Leu	0.98	1.03	1.01	1.02	1.29	0.95	1.02	1.08	0.95	0.98	1.03	9.62
Val	0.77	0.71	0.78	0.81	0.92	0.73	0.75	0.8	0.71	0.73	0.77	8.19
Arg	2.09	1.88	1.74	1.95	2.26	1.5	1.71	1.97	1.8	1.79	1.87	11.39
His	0.69	0.44	0.61	0.75	0.86	0.45	0.48	0.52	0.44	0.45	0.57	26.57
Phe	0.96	0.90	0.95	0.96	1.18	0.83	0.92	0.98	0.87	0.89	0.94	10.08
Nonessential AA												
Ala	0.77	0.73	0.72	0.78	0.88	0.72	0.81	0.9	0.66	0.67	0.76	10.60
Asp	1.67	1.69	1.62	1.77	1.95	1.54	1.67	1.75	1.55	1.58	1.68	7.33
Cys	0.14	0.06	0.08	0.11	0.17	0.06	0.07	0.07	0.07	0.07	0.09	41.90
Glu	3.66	3.63	3.35	3.96	4.55	3.22	3.69	3.84	3.39	3.46	3.68	10.41
Gly	0.75	0.75	0.73	0.79	0.91	0.68	0.82	1.25	0.69	0.70	0.81	21.10
Pro	0.75	0.50	0.80	0.87	0.99	0.66	0.73	0.96	0.62	0.63	0.75	20.86
Ser	1.03	0.83	0.77	0.87	1.00	0.7	0.76	0.77	0.72	0.74	0.82	14.01
Tyr	0.52	0.56	0.52	0.51	0.67	0.28	0.35	0.37	0.33	0.33	0.44	28.85
Total AA	16.88	15.62	15.80	17.26	20.25	14.28	15.90	17.38	14.82	15.10	16.33	10.53

**Table 4:** Ingredient composition of diets in Experiment 2 (as-fed basis).

Ingredients	Basal Diet	FCSM Diet ^1^
Corn	70.10	55.70
Soybean meal	27.20	21.60
Fermented cottonseed meal	-	20.00
Calcium hydrogen phosphate	1.00	1.00
Limestone	0.90	0.90
Sodium chloride	0.30	0.30
Vitamin and mineral premix ^2^	0.50	0.50
Total	100.00	100.00

^1^ Ten samples of fermented cottonseed meal (FCSM) were included in the diets at an identical substitution ratio. ^2^ Same as Table 2.

**Table 5:** Chemical composition of diets in Experiment 2 (as-fed basis).

Item	DM %	CP %	EE %	Ash %	CF %	NDF %	ADF %	GE MJ/kg
1	88.50	27.68	2.51	6.02	5.23	26.08	8.87	15.22
2	88.36	25.63	2.85	7.71	7.47	29.67	10.55	15.37
3	87.09	30.02	2.74	6.22	5.31	26.15	8.02	16.14
4	88.79	29.59	4.01	6.08	5.19	25.63	7.71	15.50
5	88.04	32.84	3.97	5.98	3.88	22.08	5.39	16.35
6	89.02	24.82	4.76	6.58	4.19	41.40	5.70	18.14
7	88.51	25.11	4.58	7.15	2.31	34.00	4.80	18.38
8	88.07	25.93	4.02	6.46	3.27	35.40	8.00	18.30
9	88.12	25.72	4.48	6.91	2.60	39.10	7.60	18.58
10	87.88	24.08	4.55	6.96	2.15	37.10	7.20	18.64
Mean	88.24	27.14	3.85	6.61	4.16	31.66	7.38	17.06
Max	89.02	32.84	4.76	7.71	7.47	41.40	10.55	18.64
Min	87.09	24.08	2.51	5.98	2.15	22.08	4.80	15.22
CV %	0.61	10.40	21.82	8.61	40.27	20.85	23.32	8.59

**Table 6:** Composition of fermented cottonseed meal (as-fed basis, %).

Items	FCSM ^1^ No.										Mean	CV %
	1	2	3	4	5	6	7	8	9	10		
DM	92.02	91.65	89.33	93.40	90.22	90.39	91.15	89.98	89.68	88.92	90.67	1.52
GE (MJ/kg)	18.22	15.25	17.93	17.22	17.55	18.21	18.76	18.27	18.27	18.11	17.78	5.54
CP	49.02	45.34	50.25	47.83	55.23	41.1	47.29	45.51	42.97	44.06	46.86	8.65
EE	0.87	1.01	0.96	1.47	1.38	1.90	1.40	1.68	1.60	1.30	1.36	24.46
Ash	7.43	7.85	7.83	7.13	7.08	7.39	6.64	7.04	7.38	7.81	7.36	5.4
CF	11.66	19.47	12.48	10.52	8.63	10.82	6.46	9.37	9.30	8.30	10.7	33.08
NDF	26.85	39.31	32.69	28.60	21.82	24.89	23.88	25.77	26.98	26.90	27.77	17.93
ADF	17.53	25.18	16.94	15.67	14.30	15.68	12.00	15.30	12.19	15.37	16.02	22.99
TDF	32.74	34.30	34.06	32.01	40.40	34.56	30.80	30.13	38.81	37.05	34.49	9.77
IDF	29.86	31.95	29.33	28.12	19.18	30.64	28.61	27.45	34.66	33.09	29.29	14.39
SDF	2.84	2.30	4.76	3.91	21.20	3.96	2.20	2.63	4.11	3.95	5.19	109.8
TS	0.46	1.73	0.65	0.63	0.74	0.65	0.64	0.69	0.61	0.68	0.75	47.14

^1^ The sources of the fermented cottonseed meal (FCSM) samples are provided in Table 1.

**Table 7:** Amino acid composition of fermented cottonseed meal (as-fed basis).

Items	FCSM ^1^ No.										Mean	CV %
	1	2	3	4	5	6	7	8	9	10		
Essential AA%												
Lys	1.43	1.18	1.37	1.42	1.84	1.52	1.49	1.46	1.48	1.45	1.46	11.11
Met	0.45	0.51	0.44	0.50	0.62	0.42	0.51	0.44	0.43	0.52	0.48	12.60
Thr	1.45	1.38	1.53	1.49	1.80	1.33	1.44	1.41	1.36	1.43	1.46	9.08
Trp	0.54	0.39	0.53	0.51	0.63	0.45	0.62	0.63	0.67	0.63	0.56	16.32
Ile	1.54	1.43	1.60	1.53	1.85	1.35	1.44	1.48	1.33	1.35	1.49	10.42
Leu	2.52	2.64	2.59	2.63	3.33	2.46	2.62	2.79	2.46	2.55	2.66	9.59
Val	1.97	1.83	2.01	2.08	2.37	1.88	1.95	2.05	1.82	1.89	1.99	8.14
Arg	5.37	4.84	4.48	5.03	5.82	3.84	4.42	5.07	4.63	4.60	4.81	11.45
His	1.77	1.13	1.56	1.92	2.21	1.15	1.24	1.35	1.13	1.18	1.46	26.28
Phe	2.46	2.32	2.45	2.48	3.04	2.12	2.40	2.52	2.24	2.31	2.43	10.10
Nonessential AA%												
Ala	1.97	1.88	1.85	2.02	2.26	1.82	2.11	2.33	1.76	1.73	1.97	10.45
Asp	4.31	4.36	4.18	4.55	5.01	4.05	4.28	4.49	4.02	4.05	4.33	6.95
Cys	0.36	0.16	0.21	0.28	0.45	0.16	0.20	0.16	0.17	0.18	0.23	42.88
Glu	9.42	9.35	8.61	10.19	11.72	8.31	9.52	9.86	8.73	8.88	9.46	10.41
Gly	1.92	1.93	1.88	2.04	2.33	1.77	2.15	3.27	1.81	1.82	2.09	21.43
Pro	1.94	1.29	2.05	2.23	2.56	1.72	1.86	2.47	1.58	1.64	1.93	20.81
Ser	2.66	2.13	1.98	2.25	2.58	1.84	2.06	2.02	1.85	1.93	2.13	13.46
Tyr	1.35	1.43	1.33	1.31	1.73	0.73	0.88	0.96	0.87	0.86	1.15	28.53

^1^ The sources of the fermented cottonseed meal (FCSM) samples are provided in Table 1.

**Table 8:** Mineral content of fermented cottonseed meal samples (as-fed basis).

Items	Ca %	P %	Na %	K %	Mg %	Cu mg/kg	Fe mg/kg	Zn mg/kg	Mn mg/kg	I mg/kg	Se mg/kg
1	0.23	0.98	0.26	1.52	0.67	7.24	463.38	43.72	50.23	5.88	0.11
2	0.57	0.52	0.19	1.17	0.42	3.58	2307.71	30.59	78.78	6.61	0.43
3	0.29	1.43	0.04	1.54	0.61	8.19	418.19	52.31	27.56	5.73	0.12
4	0.31	1.06	0.06	1.31	0.65	7.72	650.80	55.87	36.12	6.49	0.30
5	0.24	1.23	0.13	1.46	0.58	6.82	529.46	80.45	113.67	6.97	0.19
6	0.20	0.77	0.05	1.43	0.64	7.35	1040.27	54.50	28.61	4.87	0.15
7	0.33	0.72	0.06	1.20	0.62	8.84	318.55	58.84	42.76	4.46	0.13
8	0.22	0.83	0.28	1.28	0.67	6.76	535.92	44.78	33.53	3.53	0.11
9	0.21	0.93	0.02	1.49	0.78	8.80	796.14	60.03	30.78	4.80	0.14
10	0.22	0.73	0.03	1.45	0.31	9.00	779.63	62.66	31.75	3.94	0.16
Mean	0.28	0.92	0.11	1.39	0.60	7.43	784.01	54.38	47.38	5.33	0.18
CV %	39.23	29.13	87.36	9.70	22.65	21.37	73.47	24.38	58.78	22.21	56.02

**Table 9:** Anti-nutritional factors content in fermented cottonseed meal samples (as-fed basis).

Item	Phytic Acid %	FG ^1^ mg/g	Tannin mg/g
1	0.44	0.40	11.30
2	1.55	0.32	12.64
3	0.76	0.22	14.22
4	0.48	0.24	13.61
5	0.61	0.13	12.05
6	0.45	0.31	12.13
7	0.47	0.29	13.48
8	1.11	0.53	11.27
9	0.44	0.46	15.89
10	1.20	0.39	10.83
Mean	0.75	0.33	12.74
CV %	53.00	36.20	12.31

^1^ FG = Free Gossypol.

**Table 10:** Standardized ileal digestibility of amino acids in FCSM samples (%).

Item	FCSM ^1^ No.										Mean	SEM	*p*-Value	IAA ^2^
	1	2	3	4	5	6	7	8	9	10				
Essential AA														
Lys	68.13	65.41	60.01	68.81	65.11	68.92	64.36	62.89	67.2	68.2	65.90	4.71	0.65	2.68
Met	77.02	76.87	74.79	77.61	75.36	81.78	74.48	79.04	78.09	78.34	77.34	3.81	0.74	0.53
Thr	70.35 ^a^	68.74 ^a^	68.50 ^a^	70.69 ^a^	70.00 ^a^	70.07 ^a^	69.70 ^a^	59.97 ^b^	72.02 ^a^	60.94 ^b^	68.10	2.37	<0.01	2.72
Trp	71.24	73.62	70.25	70.39	73.55	73.05	72.38	72.76	73.51	70.43	72.12	2.06	0.51	0.68
Ile	70.21	74.84	70.13	70.59	68.29	74.26	72.56	73.64	73.24	76.25	72.40	2.03	0.06	1.60
Leu	76.57	78.96	72.15	77.65	76.65	77.68	78.60	76.83	76.24	78.08	76.94	2.36	0.26	3.46
Val	74.99 ^ab^	76.64 ^ab^	68.60 ^c^	78.16 ^a^	76.60 ^ab^	76.34 ^ab^	77.26 ^ab^	73.98 ^b^	73.76 ^b^	78.65 ^a^	75.50	1.78	<0.01	2.86
Arg	91.62	88.88	87.25	88.98	91.62	90.29	91.39	88.52	90.95	90.18	89.97	1.61	0.11	3.93
His	81.74	77.73	80.32	76.69	77.71	73.47	77.73	75.67	78.59	76.74	77.64	3.79	0.67	1.82
Phe	83.17	83.51	80.63	83.07	82.59	83.36	82.61	83.22	79.17	84.82	82.61	1.83	0.17	1.81
Nonessential AA														
Ala	73.66 ^ab^	71.3 ^ab^	66.88 ^b^	72.42 ^ab^	70.18 ^b^	74.84 ^ab^	73.83 ^ab^	67.11 ^b^	78.69 ^a^	71.92 ^ab^	72.08	3.47	0.048	2.90
Asp	80.00	80.94	78.51	79.76	78.79	78.86	79.65	78.07	78.00	81.49	79.41	2.77	0.95	5.91
Pro	82.72 ^abc^	82.81 ^abc^	81.51 ^bc^	86.26 ^ab^	83.75 ^abc^	89.57 ^a^	86.00 ^ab^	76.38 ^cd^	72.80 ^d^	80.29 ^bc^	82.21	3.49	<0.01	11.47
Cys	72.11	75.42	68.53	75.65	73.3	78.11	72.17	77.36	80.16	76.34	74.91	4.14	0.23	0.60
Glu	86.73	86.88	86.74	86.98	87.04	85.56	87.59	85.84	87.73	87.82	86.89	1.98	0.98	7.39
Gly	79.74	80.59	78.17	76.91	78.58	76.95	78.70	75.15	78.99	77.62	78.14	3.18	0.88	6.88
Ser	78.30	74.71	72.60	76.39	78.31	77.28	77.11	77.29	76.18	77.71	76.59	2.87	0.66	2.39
Tyr	74.18	71.62	69.24	75.84	74.08	76.47	72.44	72.93	75.29	72.31	73.44	2.83	0.33	0.75
TAA	81.31	81.04	78.35	81.08	80.65	81.19	81.34	71.61	80.86	81.52	79.89	4.14	0.39	66.20

^a–d^ Means in the same column with different superscript letters are significantly different at *p* < 0.05. ^1^ The sources of the fermented cottonseed meal (FCSM) samples are provided in Table 1. ^2^ **IAA**, Ileal endogenous losses of AAs (g/kg DMI).

**Table 11:** DE and ME in 10 fermented cottonseed meal for growing pigs (dry matter basis).

Item ^1^	DE MJ/kg	ME MJ/kg	ME/DE
1	10.44	9.42	0.90
2	9.69	8.55	0.88
3	11.28	11.01	0.98
4	9.98	9.63	0.96
5	12.39	11.58	0.93
6	12.65	11.84	0.94
7	10.73	10.54	0.98
8	12.07	11.33	0.94
9	12.13	11.76	0.97
10	11.66	11.19	0.96
Mean	11.30	10.68	0.94
CV %	9.24	10.51	3.54

^1^ DE, digestibility energy; ME, metabolizable energy.

**Table 12:** Prediction of DE and ME of fermented cottonseed meal from chemical composition in growing pigs (dry matter basis).

Item	Linear Regression Equations	R^2^	RMSE	*p*-Value
1	DE = 0.47 × GE + 3.25	0.44	0.83	0.04
2	DE = 0.53 × GE + 0.10 × SDF + 1.70	0.72	0.63	0.01
3	ME = 1.03 × DE − 0.94	0.91	0.35	<0.01
4	ME = 0.72 × GE + 2.51 × P − 3.89	0.87	0.47	0.001

DE, digestibility energy; ME, metabolizable energy; RMSE, root mean square error.

## Data Availability

The original contributions presented in the study are included in the article. Further inquiries can be directed to the corresponding authors.

## Abbreviations

The following abbreviations are used in this manuscript:

FCSM	Fermented Cottonseed Meal
CSM	Cottonseed Meal
DE	Digestible Energy
ME	Metabolizable Energy
SID	Standardized Ileal Digestibility
AID	Apparent Ileal Digestibility
AA	Amino Acids
CP	Crude Protein
DM	Dry Matter
GE	Gross Energy
FG	Free Gossypol
CV	Coefficient of Variation
IAA	Ileal endogenous losses of AAs
SCFAs	Short-Chain Fatty Acids
IMF	Intramuscular Fat
